# Influence of Attention Control on Implicit and Explicit Emotion Processing of Face and Body: Evidence From Flanker and Same-or-Different Paradigms

**DOI:** 10.3389/fpsyg.2019.02971

**Published:** 2020-01-21

**Authors:** Viola Oldrati, Alessandra Bardoni, Geraldina Poggi, Cosimo Urgesi

**Affiliations:** ^1^Scientific Institute, IRCCS E. Medea, Bosisio Parini, Lecco, Italy; ^2^Laboratory of Cognitive Neuroscience, Department of Languages and Literatures, Communication, Education and Society, University of Udine, Udine, Italy; ^3^Scientific Institute, IRCCS E. Medea, San Vito al Tagliamento, Pordenone, Italy

**Keywords:** face expression, body expression, emotion, implicit processing, top–down control, bottom–up interference

## Abstract

Many existing findings indicate that processing of emotional information is pre-attentive, largely immune from attentional control. Nevertheless, inconsistent evidence on the interference of emotional cues on cognitive processing suggests that this influence may be a highly conditional phenomenon. The aim of the present study was twofold: (1) to examine the modulation of attention control on emotion processing using facial expressions (2) explore the very same effect for emotional body expressions. In Experiment 1, participants performed a Flanker task in which they had to indicate either the emotion (happy/fearful) or the gender of the target stimulus while ignoring the distracting stimuli at the side. We found evidence for intrusion of the emotional dimension of a stimulus in both the emotion and gender discrimination performance, thus when either task-relevant or task-irrelevant. To further explore the influence of attention control mechanisms, in Experiment 2 participants performed a same-or-different judgment task in which they were asked to pay attention to both the central and lateral stimuli and indicated whether the central stimulus matched the lateral for emotion or gender. Results showed that emotional features exerted an influence at an implicit level (i.e., during gender judgments) for bodies only. Gender features did not affect emotional processing in either experiments. To rule out the possibility that this effect was driven by postural rather than emotional features of fearful vs. happy stimuli, a control experiment was conducted. In Experiment 3, bodies with an opening/up-ward or closing/down-ward posture but with no emotional valence were presented. Results revealed that the body posture did not influence gender discrimination. Findings suggest that the emotional valence of a face or body stimulus can overpass attention filtering mechanisms, independently from the level of attentional modulation (Experiment 1). However, broadening the focus of attention to include the lateral stimuli led emotional information to intrude on the main task, exerting an implicit, bottom–up influence on gender processing, only when conveyed by bodies (Experiment 2). Results point to different mechanisms for the implicit processing of face and body emotional expressions, with the latter likely having role on action preparation processes.

## Introduction

Adapting in a complex and dynamic environment requires the ability to remain oriented to an ongoing task and, at the same time, to direct attention to salient incoming stimuli even when they are not relevant for that task. While proactive mechanisms of attention, commonly called top–down, are deployed to maintain cognitive resources on ongoing tasks, reactive, bottom–up processes are driven by input characterized by novelty or saliency, such as emotional stimuli. The interplay between top–down and bottom–up mechanisms has been investigated with different methods (Park and Thayer, 2014; John et al., 2016; Müsch et al., 2017; Shechner et al., 2017) and in different populations, including healthy individuals and individuals in different life span with neuropsychiatric disorders (Macnamara and Proudfit, 2014; Klein et al., 2017; Mackiewicz et al., 2017; Abend et al., 2018). These studies have approached the conclusion that top–down and bottom–up mechanisms mutually influence each other in a highly conditional way (Kim et al., 2004; Pessoa et al., 2006).

One goal of the study of the interaction between attention control and emotional saliency is to weight the modulatory impact of the main ongoing task (i.e., the task at hand, which recruits the majority of cognitive resources), the affective nature of the distracting stimuli (e.g., valence, arousal and category) and the individual (clinical) characteristics of the participants (e.g., affective disorders). Overall, extensive lines of research confirmed that emotional stimuli capture attention to a greater extent than neutral stimuli at both behavioral and neural levels. At a behavioral level, stimuli charged with emotional salience have been demonstrated to elicit quicker responses in visual search paradigms using pictures of snakes/spiders among flowers/mushrooms (Ohman et al., 2001), angry/frightened faces among neutral or happy faces (Lobue, 2009; Haas et al., 2016), as well as unpleasant scenarios among neutral or pleasant scenarios (Astudillo et al., 2018). Many of these studies also indicate that stimuli with a negative valence may tap attention more than positive stimuli, suggesting that emotional valence, more than stimulus relevance, is crucial for the automatic capture of attention. The expression “negativity bias” originates from a consistent body of results stressing the importance of threat-related valence over non-threat-related contents (Carretié et al., 2009; Carretié, 2014). Nevertheless, this notion needs to be reconciled with results questioning the prioritized processing of threat-related stimuli. There is evidence stressing that the allocation of attention to emotionally salient stimuli is not automatic, but it is rather conditional to the relevance of the emotional dimension for the top–down instructions of the ongoing task (Barratt and Bundesen, 2012; Victeur et al., 2019). Other studies have argued that the “priority” dedicated to threat-related stimuli may derive from (low-level) perceptual characteristics of stimuli (e.g., visible teeth or direct eye gaze in case of angry faces) rather than from (high-level) configurational properties of the emotional expression (Horstmann and Bauland, 2006; Cooper et al., 2013). In a similar vein, evidence has been gathered around the modulatory role of the level of arousal in emotion information processing, where stimulus intensity rather than valence is considered as the key factor that drives attentional resources (Schimmack, 2005; Padmala et al., 2018). In sum, the bottom–up processing of the emotional salience, threat-related valence and arousal of stimuli seems to automatically capture attention at different extent according to the top–down requirements of the main task.

At a neural level, experimental research kept confirming that the interplay between attentional control and processing of emotional information is indeed a conditional phenomenon. The attentional competition elicited by top–down prioritization of task-related stimulus processing and automatic stimulus saliency is thought to recruit separate yet complementary brain circuits according to context demands. These circuits involve prefrontal regions, associated with attentional and complex cognitive operations, and a subcortical thalamo-amygdala pathway, which is thought as a privileged neural candidate for threat detection mechanism (Bishop, 2008). Functional magnetic resonance imaging (fMRI) studies showed that the differential amygdala response to emotional vs. neutral stimuli disappears when the cognitive load of the main task increases (Pessoa et al., 2002, 2005). In a similar vein, while attended emotional stimuli elicit greater amygdala activation as compared to attended neutral stimuli, the differential amygdala response to the two types of stimuli disappears when they are not attended to. This indicates that attentional modulation may exert a substantial influence on the pre-attentive mechanisms that underlie the automatic processing of salient stimuli (Dolan and Vuilleumier, 2003).

Among the different environmental stimuli, the processing of social-cues like faces or bodies is fundamental for our survival. Indeed, despite a heterogeneity in research methods, the automatic processing of facial (and, less extensively, bodily) emotion expressions has been the focus of several previous studies. Overall, these studies have pointed to the automatic allocation of attention to emotional face (or body) expressions even when they were presented as distractors in tasks that focused attentional resources to non-emotional target stimuli, such as letter or color detection tasks (Nordström and Wiens, 2012; Sussman et al., 2013). However, since the interplay between top–down and bottom–up mechanisms is modulated by the requirements of the ongoing tasks and by the type of stimulus saliency, investigating how the processing of facial or bodily emotional expressivity interacts with the processing of other features of the same stimuli seems relevant to dissociate the role of stimulus category (e.g., faces vs. objects) and stimulus features (e.g., emotion vs. gender) in attention allocation. Indeed, visual attention can be allocated to an object falling into a specific region of space (space-based attention) as well as to a specific feature of that object (feature-based attention; Maunsell and Treue, 2006). In this vein, studying the interaction between emotion and gender may represent an interesting opportunity, since both dimensions shape the way we interact in social environments (Harris et al., 2016).

The human visual system is highly sensitive to both emotion (Meeren et al., 2005) and gender cues (Bruce et al., 1993; Johnson and Tassinary, 2005), either conveyed by faces or bodies. Furthermore, the perception of these two salient cues seems highly interdependent, as shown by the interference exerted by variations of one dimension (i.e., gender) while recognizing the other (e.g., emotion; the so-called Garner interference effect; Gilboa-Schechtman et al., 2004; Atkinson et al., 2005; Becker, 2017; but see Gandolfo and Downing, 2019). This reveals the difficulties of the attentional control system in filtering out gender or emotional cues when the main task is focused on emotion or gender, respectively. In a recent behavioral study, the authors adopted a flanker paradigm using computer-generated faces and asked participants to indicate the emotion or the gender of the target stimulus, which could either match or not two flanker stimuli appearing at the side (Zhou and Liu, 2013). Crucially, the congruency between the target and the flanker could refer to the task-relevant (e.g., emotion congruency in the emotion task and gender congruency in the gender task) or the task-irrelevant (e.g., emotion congruency in the gender task and gender congruency in the emotion task) dimension, thus allowing the authors to directly asses the effect of emotional conflict at different levels of attention modulations. Results indicated that the incongruence of emotional (or gender) features between target and flanker interfered with emotion (or gender) discrimination especially when task-relevant, suggesting the modulation of top–down regulation mechanisms on both emotional and non-emotional feature incongruences. The researchers then applied the same paradigm coupled with the recording of ERPs and found that emotional conflict, accompanied by the peaking of the N200 component, was more pronounced when task-relevant than when task-irrelevant (Zhou et al., 2016). Nevertheless, the conflict signaled by the cortical component was observed to rise earlier for emotional incongruence than for gender incongruence. In this regard, the authors interpreted this anticipation as an index of a pre-attentive processing bias in favor of emotional over non-emotional features. Going toward a similar direction, a recent study observed an enhanced N170 component for emotional as compared to neutral face expressions when participants were asked to recognize the gender of laterally presented faces. Interestingly, this effect disappeared when participants were asked, rather than responding on a dimension of the faces, to detect a missing pixel of the central fixation cross, providing further evidence of a modulation of task demands on emotional features interference (Burra and Kerzel, 2019).

In light of the latest literature, the present work was aimed at replicating previous findings (Zhou and Liu, 2013) that either emotion or gender information is modulated by attentional control when task-relevant applying a similar flanker paradigm (Experiment 1). However, we also tested whether the same effects were maintained when using a same-or-different comparison task (Experiment 2). This second paradigm was selected to explore the modulatory role of visual search strategy on stimuli processing and distractor filtering and to test the modulatory role of space-based and feature-based attention allocation (Maunsell and Treue, 2006). The flanker task invites to focus the attention on the central target of a set of stimuli, which may favor space-based filtering of distractors. The same-or-different judgment task requires to expand the focus of attention to the entire set. This could hinder space-based filtering (i.e., suppressing the processing of an object based on its location) and possibly favor feature-based strategy (i.e., suppressing the processing of a specific feature of an object). This fundamental difference between the two tasks is reflected in a more pronounced center-to-periphery gradient of visual search in the flanker than in the same-or-different paradigm (Wendt et al., 2017).

A second aim was to explore the modulatory effect of attention on emotion and gender processing when conveyed not only by faces, but also by bodies. In fact, both faces and bodies provide important cues for the adaptation to social environments. Existing evidence suggests that face and body processing share similar cognitive mechanisms and neural correlates (Minnebusch and Daum, 2009 for a detailed review). For example, there is evidence that both faces and bodies trigger configural processing, defined as the perception of relations among the features of a stimulus (Reed et al., 2006). Moreover, faces and body stimuli specifically activate distinct yet adjacent and overlapping brain regions within the lateral and medial occipito-temporal cortex (Peelen and Downing, 2019). Another indication of a certain degree of similarity between the two comes from clinical studies. Patients diagnosed with prosopagnosia, an acquired disorder of face recognition, have also difficulties with the configural processing of bodies (Righart and de Gelder, 2007; Moro et al., 2012). Nevertheless, analogies from faces to bodies cannot ignore the perceptual and affective differences existing between the two classes of stimuli. As to their functional role, theoretical models suggest that faces would trigger more emphatic-related processes, while bodies processing would involve systems deputed to action observation and preparation (van de Riet et al., 2009).

To these aims, we run three experiments in which we presented a central face or body sided by two lateral faces or bodies that could match or mismatch the gender or emotion of the central stimulus. In Experiment 1, we used a typical flanker paradigm, in which attention was directed only to the central target stimulus and the influences of task-relevant and task-irrelevant features of the lateral stimuli were tested. In Experiment 2, we used a same-or-different paradigm, in which attention was directed to both central and lateral stimuli and participants had to match the central with the lateral stimuli for either gender or emotion. Finally, a third control experiment tested the specificity of the interference effects for bodily emotions as compared to neutral body postures.

## Experiment 1

In this experiment, we used a typical Flanker paradigm to test whether the attentional modulation (i.e., task relevance) of the relative influence of gender and emotional cues was comparable for face and body stimuli. We presented a central face or body target sided by two flanker faces or bodies that could match or not the central target in gender or emotion. In separate blocks, we asked participants to discriminate the gender (i.e., male or female) or the emotion (i.e., positive or negative) of the central target. In keeping with previous studies with faces (Zhou and Liu, 2013), we expected that the presentation of the two flanker stimuli should interfere with the discrimination of the gender or emotion of the central target more when they are incongruent for the task-relevant (i.e., incongruent gender for the gender task and incongruent emotion for the emotion task) than for the task-irrelevant (i.e., incongruent emotion for the gender task and incongruent gender for the emotion task) dimension. This modulation should be comparable for faces and bodies as well for gender and emotional tasks, even if emotional incongruence was expected to exert a marginal effect also on gender discrimination, in keeping with a greater resilience of emotion processing to top–down attentional control (Zhou and Liu, 2013).

### Participants

Twenty-four healthy volunteers (7 men, *M* = 28.04 *SD* = 4.15 years old) participated in Experiment 1. All participants had normal or corrected-to-normal vision. Prior to the beginning of the experiment, written informed consent was obtained from all participants. The experiment was approved by the local ethical committee and conducted in accordance with the declaration of Helsinki.

### Stimuli and Task

Experimental stimuli consisted of a total of 24 pictures of Caucasian faces and bodies. Faces were taken from the NimStim dataset (Tottenham et al., 2009). Two emotion expressions (happy and fearful) were chosen for each face, so that in total six female and six male faces (three happy and three fearful) were used. Faces whose emotion expression was recognized with an accuracy level equal or superior to 80% were selected (Tottenham et al., 2009). Both happy and fearful faces were with the mouth open, as these have been shown to be better recognized than closed-mouth expressions (Tottenham et al., 2009).

Bodies pictures were taken from a validated pool of static images depicting bodies with blurred faces in emotional whole-body movements (see Borgomaneri et al., 2012 for details). Two emotion expressions (happy and fearful) were chosen for each body image, so that in total six female and six male bodies (three happy and three fearful) were used.

**FIGURE 1:**
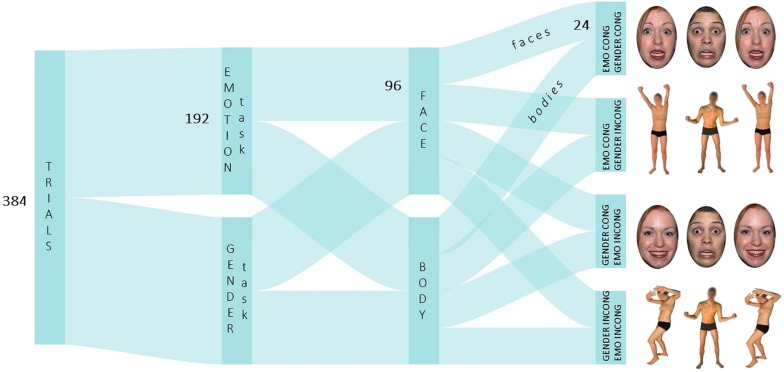
Schematic depiction of the task structure in Experiment 1 and Experiment 2.

Participants were presented with two Eriksen and Flanker tasks (Eriksen and Eriksen, 1974) focusing either on the emotion expression or on the gender of the stimuli. Each task consisted of two blocks, one presenting face stimuli and the other one presenting body stimuli (Figure 1). The order of presentation of both tasks and types of stimuli were counterbalanced between participants. Face and body stimuli were displayed on a dark background. A trial displayed an array of three stimuli, each one subtending a visual angle of 2.5° × 3.5° for faces and 2.5° × 5.6° on average for bodies, considering a viewing distance of about 60 cm from the computer screen. A visual angle of 3° wat set between the center of the target and the center of each flanker. Faces and bodies differ in terms of structure, which is closed for faces and open for bodies. Moreover, while emotion expressions do change the disposition of bodily parts within space (e.g., opening/up-ward vs. closing/down-ward posture for expressing happy vs. fearful emotions, respectively), this is not the case for faces. Given this structural difference, the width of body images including the portion of space covered by legs and arms (2.7 cm on average) was manipulated to match the width of face images (2.6 cm). Participants were asked to indicate the emotion (emotion task) or the gender (gender task) of the face/body displayed in the middle of the array (i.e., the target) while ignoring the ones displayed at the side (i.e., the flanker). Emotional and gender features across the array of stimuli were presented in four different combinations: emotion different/gender different, emotion different/gender same, emotion same/gender different, emotion same/gender same. Thus, in the emotion task, emotional features were task-relevant and gender features were task-irrelevant; conversely, in the gender task, emotional features were task-irrelevant and gender features were task-relevant. Participants provided their response with a left/right mouse click using their thumbs. Response key assignment was counterbalanced among participants. The array of stimuli appeared on the screen for 500 ms, followed by a blank screen displayed for 2,000 ms. A time-window of 500 ms for stimuli presentation has been proven sufficient to elicit an emotional congruency effect in a flanker paradigm with facial expressions (Holthausen et al., 2016). The arrival of a new trial was signaled by a fixation cross of 400–600 ms appearing in the center of the screen. Participants could provide their response from the beginning of the trial till the presentation of the fixation cross.

### Statistical Analysis and Sample-Size Calculation

Inverse efficiency scores (IES), computed by dividing mean response times by proportion of correct responses per subject and per condition, were selected as the main dependent measure. Integrating mean response times and correct responses into a single dependent variable represents a suitable option to appropriately weighs the impact of speed and accuracy, particularly in case of a negative correlation between the two measures (Bruyer and Brysbaert, 2011). For all the experiments, trials with response times below 100 ms were excluded from further analysis. A 2 × 2 × 2 × 2 ANOVA on IES was conducted with TASK (emotion vs. gender recognition), STIMULI (face vs. body) EMOTION CONGRUENCY (congruent vs. incongruent emotion) and GENDER CONGRUENCY (congruent vs. incongruent gender) as within-subjects variables.

In case of significant main or interaction effects, follow-up pairwise comparisons were executed adopting the Duncan’s Multiple Range Test. The alpha level of significance was set at 0.05 (two-tailed).

On the basis of a 2 × 2 × 2 × 2 repeated measure ANOVA on IES, considering a medium-low effect size of 0.25 – as reported in a published study using a similar paradigm (Zhou and Liu, 2013) – and applying an alpha level of 0.05, a sample of 24 participants was defined as adequate to achieve a power of 0.80 (1-Beta).

### Results

The four-way repeated-measure ANOVA on IES yielded significant main effects of TASK [*F*(1,23) = 7.8, *p* < 0.02, η^2^_p_ = 0.25], STIMULI [*F*(1,23) = 25.5, *p* < 0.0001, η^2^_p_ = 0.53] and a significant effect of EMOTION CONGRUENCY [*F*(1,23) = 4.8, *p* = 0.04, η^2^_p_ = 0.17]. Overall, participants were less efficient in emotion discrimination than gender discrimination and in processing body than face stimuli. Furthermore, they showed a tendency to be less efficient to respond to the central target when the lateral flanker had an incongruent vs. congruent emotion (mean IES in emotion congruent = 626.4 ms, SEM = 10.7 vs. incongruent = 636.5 ms, SEM = 10.8). This occurred in both the emotion-discrimination and the gender-discrimination tasks (interaction TASK × EMOTION CONGRUENCY: [*F*(1,23) < 1]), thus when the (in)congruency was either relevant or irrelevant to the task, and for both the body and the face stimuli (interaction STIMULUS × EMOTION CONGRUENCY: [*F*(1,23) < 1]). The main effect of GENDER CONGRUENCY [*F*(1,23) < 1] and any other interactions were non-significant [for all *F*(1,23) < 3, *p* > 0.1]. Accuracy and RTs among conditions are reported in Table 1.

**TABLE 1:** Mean RTs and Accuracy for each experimental condition in Experiment 1.

		**Emotion Recognition**				**Gender Recognition**			
		**Face**		**Body**		**Face**		**Body**	
		***RT (ms)***	***Acc %***	***RT (ms)***	***Acc %***	***RT (ms)***	***Acc %***	***RT (ms)***	***Acc %***
**Emotion features**	**Gender features**								
Congruent	Congruent	594.88	0.97	642.88	0.94	554.06	0.98	609.72	0.95
	Incongruent	597.14	0.97	622.66	0.94	555.14	0.98	599.31	0.93
Incongruent	Congruent	603.01	0.96	637.74	0.95	555.00	0.98	622.47	0.92
	Incongruent	600.20	0.95	635.10	0.93	546.85	0.97	608.75	0.94

### Discussion

**FIGURE 2:**
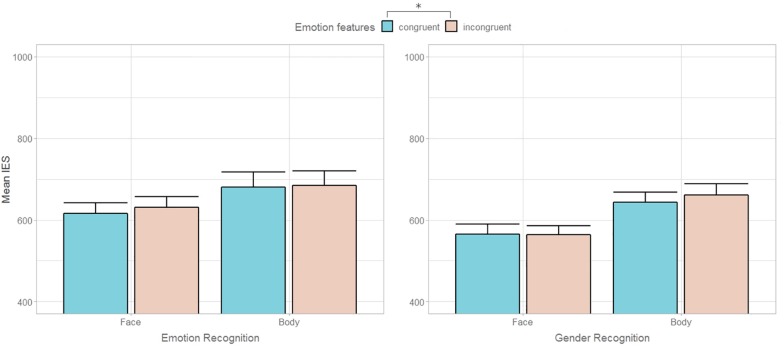
Mean inverse efficiency scores (IES) during Emotion and Gender recognition according to task-relevant **(left graph)** and task-irrelevant **(right graph)** emotional congruency between target and flanker in Experiment 1. Error bars represent + 1 SEM. The asterisk * indicates the main effect of emotion congruency (*p* < 0.05).

In this flanker task, participants were asked to recognize either the emotion (happy/fearful) or the gender of the target stimulus while ignoring the distractors, which could match or not either the task-relevant features (e.g., emotional features in emotion discrimination) or the task-irrelevant ones (e.g., gender features in emotion discrimination). The results suggested that, despite the participants had to focus on the central target and ignore the lateral flanker, the congruency between the emotional valence of the target and flanker stimuli impacted both the emotion and gender discrimination performance (Figure 2). Thus, in contrast with Zhou and Liu (2013)’s results, we found evidence for intrusion of the emotional dimension of a stimulus independently from attentional modulation. Conversely, non-emotional feature conflict (i.e., gender incongruence) did not affect performance when either relevant (i.e., gender discrimination) or irrelevant for the task at hand (Figure 2). This result is inconsistent with previous findings showing, firstly, the presence of both emotional and gender congruency effects – with participants being slower in processing incongruent trials – when features are task-relevant and, secondly, that both these effects are attenuated when congruency became task-irrelevant (Zhou and Liu, 2013). It should be noted that the lack of interaction effects between task and congruency may be attributed to a statistical power issue. In fact, one can fairly assume that increasing the number of trials from 192 to 256, as in Zhou and Liu (2013)’s work, may result not only in a significant main effect of emotion but also in a significant interaction between emotional congruency and task, which would indicate the presence of attention modulation. This notwithstanding, the authors also observed that the attenuation of the congruency effect in the task-irrelevant condition was less pronounced for emotion than gender. This finding drove them to the conclusion that, even though attentional control can exert a top–down modulation on emotion processing, yet emotional saliency can be modulated by attention resources to a lesser degree than non-emotional contents.

Then, how can the discrepancy between our findings and previous evidence be explained? In both Zhou and Liu (2013)’s and our data, gender recognition was easier than emotion recognition, thus arguing against the possibility that the asymmetry between emotion and gender conflict could be due to a mismatch in task difficulty. Furthermore, interference from automatic processing is expected to be higher for more easily encoded and, thus, more salient features (Atkinson et al., 2005; Gandolfo and Downing, 2019). In contrast, we found that the more difficult-to-encode dimension (i.e., emotion) interfered with the easier one (i.e., gender), thus making it unlikely, albeit not ruling out, that the asymmetry between emotion and gender conflict was due to differential speed of processing. A possible answer to the discrepancy between our results and those of Zhou and Liu (2013) may be found in the choice of the type of stimuli. While in the present experiment real face stimuli (and real bodies) were applied, Zhou and Liu used computer-generated faces. Despite little evidence is available on the resemblance between computer-generated and real faces, the emotion recognition of virtual (Dyck et al., 2008) or iconic (Kendall et al., 2016) and of real or photographic face images may be different. Moreover, the use of multiple identities in the present study, as opposed to the adoption of a single face identity in Zhou and Liu (2013)’s study, may explain the discrepancy between the two findings. In fact, displaying different identities presumably increased the variability of low-level features of the stimuli and may have challenged the processing of their gender.

Another possible answer may call into play a temporal factor. Zhou and Liu displayed target and flanker for 1,400 ms, while we presented them for 500 ms. Even though the mean response times reported by the researchers seem comparable to the response times measured in the present experiment, having posed a more strict time-constraint, as in this case, may have allowed only emotional conflict to be processed enough for exerting bottom-up interference on the main task. This is in keeping with the finding of an earlier modulation of ERP cortical components exerted by emotional than by non-emotional feature conflict (Zhou et al., 2016). Based on these considerations, it can be hypothesized that reducing the exposure time to stimuli and focusing on an early decoding time window may have increased the chance to observe emotional features over non-emotional features conflict, at least when participants had to focus their attention on the central target and completely ignore the objects at the side. However, the different weight of emotional or non-emotional conflict on attentional control may change when participants have to focus on both the central target and the lateral flanker to perform a gender or emotion comparison, while ignoring the irrelevant dimension (i.e., emotion or gender, respectively), thus tapping on feature-based rather the space-based attention allocation mechanisms.

## Experiment 2

Experiment 2 was aimed at examining whether expanding the focus of attention across the visual field may modulate the interplay between attentional control and emotional and non-emotional feature processing. To this aim, we used the same stimuli from Experiment 1 in a same-or-different judgment paradigm in which participants had to match the emotion or the gender of the central and lateral stimuli. Thus, while the participants’ main task focused on the task-relevant congruency of the stimuli (i.e., emotion congruency in the emotion task and gender congruence in the gender task), we tested the effects of the task-irrelevant dimension (i.e., gender congruency in the emotion task and emotion congruency in the gender task). We expected that, different from Experiment 1, both gender and emotional task-irrelevant incongruence between the lateral and central stimuli should affect the main task when task relevant. Furthermore, in keeping with Experiment 1, the same effects should be obtained for body and face stimuli.

### Participants

Twenty-four healthy volunteers (10 men, *M* = 28.6 *SD* = 5 years old) participated in Experiment 2. One participant was excluded from analyses due to below-chance accuracy in one of the tasks. All participants had normal or corrected-to-normal vision. Prior to the beginning of the experiment, written informed consent was obtained from all participants. The experiment was approved by the local ethical committee and conducted in accordance with the declaration of Helsinki.

### Stimuli, Task and Analysis

The same stimuli, task structure, procedure and data handling as in Experiment 1 were used in Experiment 2. However, in this experiment participants were asked to perform two same-or-different judgment tasks focusing either on the emotion expression or on the gender of the stimuli. Participants indicated whether the emotion (emotion task) or the gender (gender task) of the stimulus displayed in the middle of the array matched or not that of the stimuli at the sides.

### Results

A 2 (TASK, emotion vs. gender comparison) × 2 (STIMULI, face vs. body) × 2 (task-irrelevant CONGRUENCY) repeated measure ANOVA on IES yielded significant main effect of STIMULI [*F*(1,22) = 20.95, *p* < 0.001, η^2^_p_ = 0.49], significant interaction effects of TASK^∗^STIMULI [*F*(1,22) = 10.52, *p* < 0.005, η^2^_p_ = 0.32] and of TASK^∗^STIMULI^∗^CONGRUENCY [*F*(1,22) = 8.09, *p* < 0.01, η^2^_p_ = 0.27]. No other significant main nor interaction effect emerged. The analysis showed that participants were less efficient in processing bodily stimuli, similarly to what emerged in Experiment 1, and that this effect emerged during the gender congruency comparison (mean IES of face = 1101.0 vs. body = 1385.3 vs.; *p* < 0.001). Indeed, during the emotion congruency comparison no difference between type of stimuli emerged (face = 1215.3 body = 1252.6; *p* < 0.41). The three-way interaction revealed that participants were less efficient in processing body gender congruency in emotional (i.e., task-irrelevant) incongruent trials (emotion congruent = 1345.9 vs. emotion incongruent = 1424.6; *p* < 0.02). Conversely, no difference emerged for face gender congruency processing according to emotional features congruency (emotion congruent = 1103.6 vs. emotion incongruent = 1098.4; *p* < 0.77). No other significant difference emerged during emotion congruency comparison according to gender feature congruency for face expressions (gender congruent = 1201.5 vs. gender incongruent = 1229.1; *p* < 0.33) nor body expressions (gender congruent = 1266.6 vs. gender incongruent = 1238.7; *p* < 0.21). Accuracy and RTs among conditions are reported in Table 2.

**TABLE 2:** Mean RTs and Accuracy for each experimental condition in Experiment 2.

	**Emotion Comparison**					**Gender Comparison**			
	**Face**		**Body**			**Face**		**Body**	
	***RT (ms)***	***Acc %***	***RT (ms)***	***Acc %***		***RT (ms)***	***Acc %***	***RT (ms)***	***Acc %***
**Gender features**					**Emotion features**				
Congruent	1033.05	0.87	1112.01	0.89	Congruent	1004.48	0.90	1070.40	0.80
Incongruent	1041.58	0.86	1109.65	0.90	Incongruent	1004.87	0.89	1094.69	0.78

### Discussion

**FIGURE 3:**
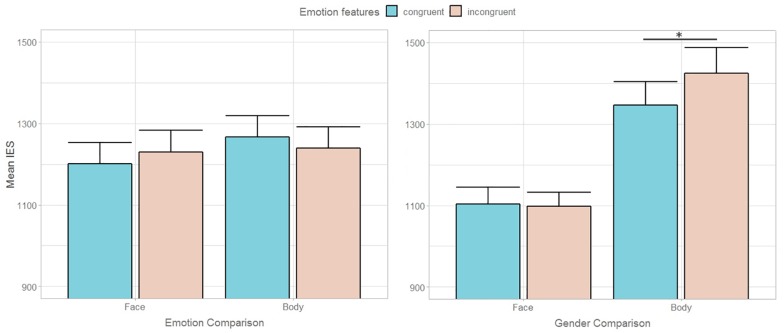
Mean inverse efficiency scores (IES) during Emotion and Gender comparison according to task-relevant **(left graph)** and task-irrelevant **(right graph)** emotional congruency between central and lateral stimuli in Experiment 2. Error bars represent + 1 SEM.

Results of Experiment 2 showed that, on the one hand, even broadening the focus of attention to include the lateral stimuli did not trigger a bottom-up interference of non-emotional feature conflict (i.e., gender incongruence) on the main task. It is worth noting that, in Experiment 1, gender recognition was overall easier than emotion recognition, leaving open the possibility that the asymmetry in the relative influence of gender and emotional conflict could arise from different task difficulty. Conversely, in Experiment 2, the need to compare the central and the flanker stimuli increased the task demands for both the gender and the emotion features, and no difference between the two tasks emerged. This clears out the even remote scenario that greater interference effects are exerted by more difficult to encode features. On the other hand, results showed a differential influence of emotional features on an implicit level between faces and bodies. Indeed, while participants were less efficient in discriminating the gender in emotional incongruent than congruent trials when processing bodies, this difference was not found in response to faces (Figure 3). Alternative explanations may account for this result. First of all, it cannot be excluded that hampered gender comparison in emotional incongruent trials was driven by “intrinsically” postural, rather than emotional features. A static body is generally recognized as fearful in presence of specific anatomical characteristics, such as abdominal rotation, shoulders ad/abduction or backwards transfer of body weight (Coulson, 2004). As morphologic, sexually dimorphic cues as the waist-to-hip ratio or the shoulders’ width have been demonstrated to be crucial for gender categorization (Lippa, 1983; Johnson and Tassinary, 2005), one can fairly assume that a bodily posture partially hiding or distorting these cues may hamper the discrimination of its gender. Therefore, the possibility that fearful, as opposed to happy, expressions may have interfered with gender comparison for the distortion of relevant sexually dimorphic cues rather than for their emotional valence cannot be dismissed. Moreover, the different degree of variation in morphologic features between facial and bodily emotional expressions, more pronounced in the latter, may explain the differential effect between the two types of stimuli.

## Experiment 3

A third control experiment was carried out specifically to examine the possibility that postural rather than emotional features may have affected gender comparison when task-irrelevant in Experiment 2. Here, participants were presented with images of bodies with an opening/up-ward or closing/down-ward posture with no emotional valence, selected with the purpose of resembling the approaching or avoidant movements associated with the expression of happiness or fear respectively. If the effect observed in Experiment 2 was likely to be driven by the emotional salience of the stimuli, then perceptual characteristics of body postures should not interfere with gender comparison processing. Conversely, a similar pattern of results as in Experiment 2 was expected if posture, rather than emotional valence, explained the task irrelevance interference in Experiment 2.

### Participants

Twenty-four healthy volunteers (8 males, *M* = 29 *SD* = 4.2 years old) participated in this experiment. All participants had normal or corrected-to-normal vision. Prior to the beginning of the experiment, written informed consent was obtained from all participants. The experiment was approved by the local ethical committee and conducted in accordance with the declaration of Helsinki.

### Stimuli, Task and Analysis

**FIGURE 4:**
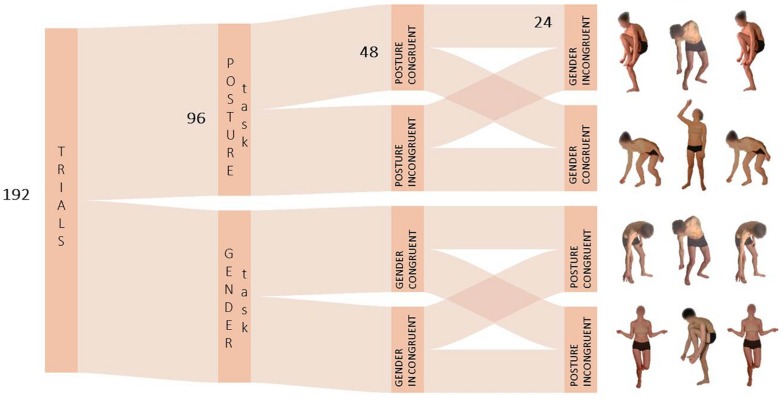
Schematic depiction of the task structure in Experiment 3. In the Posture Task, the feature to be attended was the opening/up-ward vs. closing/down-ward movement of bodies with no emotional valence.

The same task structure, procedure and data handling of Experiment 1 and 2 were used in Experiment 3. However, in this control experiment only blocks with body images were used. Here, participants were asked to perform two same-or-different judgment tasks focusing either on the posture or on the gender of the stimuli. Two types of whole-body movements without emotional valence were selected: opening/up-ward and closing/down-ward body movements (Figure 4). Selected stimuli resembled the approaching or avoidant dynamics generally associated with the expression of happiness and fear respectively. Participants indicated whether the type of movement or the gender of the target stimulus matched with the stimuli at the side according to the task-relevant feature to be attended.

### Results

A 2 (TASK, posture vs. gender comparison) × 2 (task-irrelevant CONGRUENCY) repeated measure ANOVA on IES yielded significant main effect of TASK [*F*(1,23) = 51.91, *p* < 0.00001, η^2^_p_ = 0.69], indicating low efficiency in making gender as compared to postural features judgments. No other significant main nor interaction effect emerged. Accuracy and RTs among conditions are reported in Table 3.

**TABLE 3:** Mean RTs and Accuracy for each experimental condition in Experiment 3.

	**Gender**			**Posture**	
	**Comparison**			**Comparison**	
	***RT (ms)***	***Acc %***		***RT (ms)***	***Acc %***
**Posture features**			**Gender features**		
Congruent	1153.63	0.77	Congruent	932.82	0.92
Incongruent	1158.49	0.78	Incongruent	910.62	0.91

### Discussion

**FIGURE 5:**
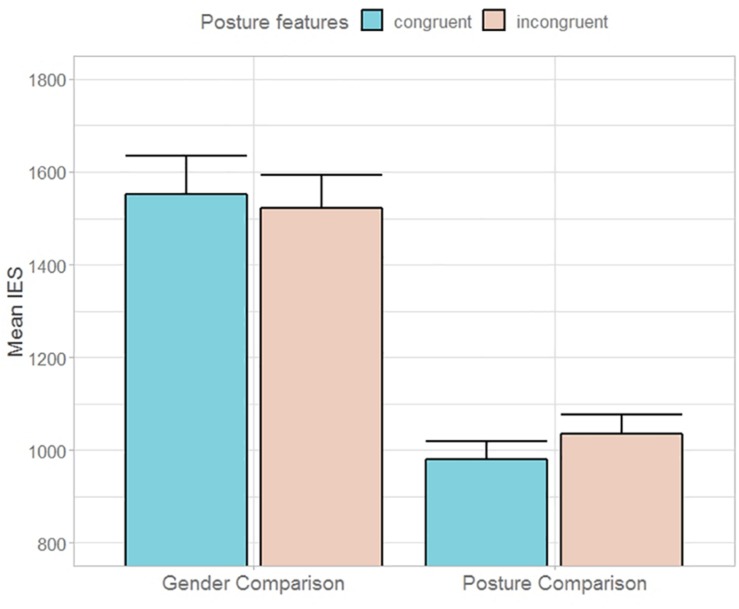
Mean inverse efficiency scores (IES) during Gender and Posture comparison according to task-irrelevant postural congruency between central and lateral stimuli in Experiment 3. Error bars represent + 1 SEM.

Results of Experiment 3 revealed that body posture did not influence gender comparison, suggesting that findings of Experiment 2 may indeed reflect an intrusion of information of emotional nature on the main task. In fact, even though opening/up-ward and closing/down-ward bodies were comparable to happy/fearful bodily expressions in terms of visibility of perceptual sexual cues, participants did not perform poorly in postural incongruent trials as compared to congruent trials (Figure 5). Simply put, emotional information and not postural features of body expressions could be held responsible for having interfered with gender comparison in Experiment 2. Conversely, task-irrelevant gender congruency did not affect posture comparison here, as it did not affect emotion comparison in Experiment 2.

## General Discussion

In the present study we tested the role of attentional modulation on the processing of bodily and facial emotion and gender features. We found that, while attention was focused on a central stimulus (flanker task; Experiment 1), the emotion features of two unattended lateral faces or bodies intruded in the main task, when either task relevant or task irrelevant. Conversely, the distracting gender features were successfully filtered out, in both the task relevant or task-irrelevant conditions, reducing the impact of ongoing mechanisms of conflict resolution. This suggests that the attentional control system can suppress distracting information that is void of any emotional valence via space- and/or feature-based filtering mechanisms. It can be speculated that filtering of flanker gender in Experiment 1 underpinned a space-based attention mechanism, confining attentional resources to a specific region of interest, selecting the stimuli displayed in that region and hindering this way the erroneous selection of distractors. Yet, our findings suggest that the emotional valence of a face or body stimulus can overcome this spatial attention filtering, in keeping with the evidence of reduced extinction of controlesional emotional vs. neutral faces (Vuilleumier et al., 2002) or bodies (Tamietto et al., 2007) in brain-lesion patients.

When the focus of attention was manipulated to include the processing of lateral stimuli (same-or-different task; Experiment 2 and 3), disabling what can be referred to as a proactive control relying on space-based selection of input, only emotional expressions (Experiment 2) but not neutral postures (Experiment 3) of bodies affected the processing of their gender when task-irrelevant. However, task-irrelevant gender features of both faces and bodies and emotional features of faces were filtered out and did not intrude in the main task at hand. This may be due to the action of a feature-based filtering, operating a selection of the properties rather than (only) the location of the input. Both mechanisms were capable of an efficient filtering selection of non-emotional feature of both body and faces. However, the present findings suggest that the space-based mechanism, likely active in the Flanker task in Experiment 1, was pervious to emotional information conveyed by both faces and bodies, which both showed a comparable interference on the main task when the emotional dimension was either task relevant or irrelevant. Conversely, the feature-based mechanism was more effective in filtering out task-irrelevant emotional features of attended facial expressions, but it was pervious to task-irrelevant emotional information conveyed by body postures. This seems to point to a greater resilience of body than face emotional processing to feature-based filtering selection.

A question arises, thus, regarding the differential influence of implicit processing of emotional information between faces and bodies. At first, one may argue that bodily expressions conveyed emotion information more efficiently than faces. However, not only both types of stimuli were selected from validated databases among stimuli associated with the highest rates of recognition accuracy, but recent behavioral studies also demonstrated that the recognition rate of emotional expressions is similar for faces and bodies (de Gelder et al., 2014). Furthermore, the main effect of stimuli in both Experiments 1 and 2 may lead to consider the modulatory role of task difficulty to be at the basis of the discrepancy between faces and bodies. Indeed, participants were significantly less efficient when processing body as compared to face images. Nevertheless, behavioral indices of emotional interference have been reported at very different levels of cognitive engagement (Lim et al., 2008; Muller et al., 2008). Moreover, a meta-analytic investigation found the association between magnitude of the effect of emotional information intrusion on attention control and task difficulty to be non-significant (Carretié, 2014). Finally, while a differential performance in discriminating or matching faces and bodies was present in both Experiments 1 and 2, task-irrelevant emotional features of faces intruded in the main task in Experiment 1 but not in Experiment 2.

Alternatively, another explanation for this result calls into play the specific role of bodies in the communication of emotions. Understanding emotions expressed by other individuals is crucial to adapt our behavior in the physical and social world. Indeed, it has been suggested that emotional body images may boost the activation of brain regions involved both in emotional information processing and in the perception of our bodily experience (e.g., insula and somatosensory cortex) more than images of faces or affective pictures in general (de Gelder, 2006). This presumably occurs because, while emotional facial expression may trigger different responses, as they can signal the presence of a salient stimulus or, alternatively, invite other individuals to empathize, a body expression of emotional valence constitutes a more direct cue of how to cope with the environment. For example, compared to faces, fearful bodies provide not only information of the emotional state of the individual, but also additional information on how to cope with the threat that has been signaled through specifications of the postural schema.

Congruently, the processing of emotional cues conveyed by whole-body postures and movements implies the activation of a broad brain network, including not only regions typically involved in emotion perception, but also regions part of the motor system (Urgesi et al., 2006; de Gelder et al., 2014). Specifically, emotional bodily expressions have been demonstrated to automatically activate areas involved in action representation and preparation as compared to meaningful but emotionally neutral bodily postures (de Gelder et al., 2004). Furthermore, a motor response is elicited by bodily expressions even when presented in the blinded hemifield of patients with brain lesions (Tamietto et al., 2015) or in the unattended visual field of patients with hemispatial neglect (Tamietto et al., 2007, 2009). This piece of evidence supports that emotional body signals may be prioritized due to a processing bias, presumably because of their critical importance for survival.

Accordingly, evidence from brain stimulation studies showed altered corticospinal excitability, which might reflect action readiness to environmental input, during passive viewing of emotional images as compared to non-emotional images (Olivieri et al., 2003; Schutter et al., 2008; van Loon et al., 2010). A later brain stimulation study confirmed these findings using body images, strengthening the link between processing of emotional body expressions and action observation and preparation. Borgomaneri et al. (2012) observed a reduced cortico-spinal excitability in the left hemisphere during emotion categorization of both happy and fearful bodily expressions as compared to neutral body images. While this work did not find a differential effect of negative and positive emotion valences (but see Borgomaneri et al., 2015), it allowed controlling for the confounding effect of perceived motion in influencing early motor response, showing a differential effects for emotional and non-emotional postures despite comparable implied-motion effects. This is in keeping with our findings that emotional expressions, rather than postures, were driving the implicit, task-irrelevant interference on gender discrimination. Thus, as previously shown for affective pictures (Bannerman et al., 2009), emotional body language can capture attention automatically.

Previous research points to a prioritized processing of emotional stimuli over non-emotional ones. Nevertheless, there are studies that failed in finding a clear modulation of emotion processing on attention resources. This lack of modulation effect may be due to various factors, such as methodologic characteristics of paradigms and their ability to elicit distinct top–down regulation strategies, or type of stimuli. Accordingly, while a proactive top–down regulation was pervious to intrusion via bottom–up processing of emotional information conveyed by bodies and faces in the Flanker task used in Experiment 1, the link between emotional salience and action readiness may explain why, in Experiment 2, emotional information interfered with feature-based down-regulation mechanisms only when conveyed by bodies and not by faces.

The possible role of emotional valence and social relevance of the stimuli in attentional modulation may be relevant for exploring the mechanism of disordered attention. Indeed, compared to a consistent body of literature on the modulatory role of phobic and anxiety disorders on perceptual emotion processing (Sussman et al., 2016; Wante et al., 2016), limited evidence has been gathered so far on the influence of attention control and inhibition difficulties. A study on children and adolescents diagnosed with attention deficit and hyperactivity disorder (ADHD) showed that, during a digit categorization task, emotional distractors were associated with longer reaction time than neutral distractors in the clinical sample, while the very same difference was not observed in the control group (López-martín et al., 2013). Moreover, at an electrophysiological level this finding was accompanied with an increase in the amplitude of the event-related potential (ERP) component N200 for emotional as compared to neutral distractors, again only in the ADHD group. Likewise, other pathologies associated with attention problems may underpin an altered interaction between top–down inhibitory control and bottom-up affective processes. In a rehabilitation perspective, further research is still needed to shed a light on this interaction in abnormal neuropsychological profiles, in order to disentangle the reciprocal impact of attentional modulation on emotional processing.

## Data Availability Statement

The datasets generated for this study are available on request to the corresponding author.

## Ethics Statement

The studies involving human participants were reviewed and approved by the Ethics Committee of Scientific Institute IRCCS E. Medea, Bosisio Parini, Italy. The patients/participants provided their written informed consent to participate in this study.

## Author Contributions

VO and CU conceived and designed the experiments, and analyzed and interpreted the data. AB and GP contributed to the design of the experiments. VO performed the experiments and wrote the original draft of the manuscript. VO, AB, GP, and CU reviewed and edited the manuscript. All authors contributed to the final approval of the manuscript.

## Conflict of Interest

The authors declare that the research was conducted in the absence of any commercial or financial relationships that could be construed as a potential conflict of interest.
